# Seeing beyond white light: fluorescence for safer laparoscopic cholecystectomy

**DOI:** 10.3389/fsurg.2026.1847578

**Published:** 2026-05-19

**Authors:** Jaime López-Sánchez, Francisco José Mena, Nerea García, María Carmen Esteban, Francisco Blanco

**Affiliations:** 1 Department of General and Gastrointestinal Surgery, Hospital Universitario de Salamanca, Salamanca, Spain; 2 Biomedical Research Institute of Salamanca (IBSAL), Instituto de Investigacion Biomedica de Salamanca, Salamanca, Spain; 3 Universidad de Salamanca, Salamanca, Spain

**Keywords:** acute cholecystitis, bile duct injury, cholangiography, indocyanine green, laparoscopic cholecyctectomy

## Abstract

Laparoscopic cholecystectomy (LC) remains the standard treatment for symptomatic gallstone disease, yet bile duct injury persists as a serious and feared complication, predominantly caused by misidentification of biliary anatomy. Fluorescence-guided imaging with indocyanine green (ICG) has gained prominence as an adjunct to enhance real-time anatomical interpretation and strengthen intraoperative safety. This mini review synthesizes current evidence supporting fluorescent cholangiography in both elective and acute care settings, where inflammation, distorted anatomy, and urgent decision-making heighten surgical risk. The manuscript provides an updated overview of the technical foundations of ICG fluorescence, practical considerations for its administration, and its incorporation into modern surgical workflows. It compiles data demonstrating improved visualization of key biliary structures, and potential reductions in complications, conversion to open surgery, and operative time. The review also addresses persistent limitations, such as heterogeneity in dosing protocols, interference from hepatic background fluorescence, and reliance on specialized imaging equipment, that continue to impede universal standardization. Taken together, these findings position fluorescence imaging as a pragmatic, radiation-free modality that extends the surgeon's visual capabilities “beyond white light,” contributing to safer and more precise LC. Ongoing technological refinement and well-designed multicenter studies will be crucial to confirm its clinical benefits and define its place in routine surgical practice.

## 1 Introduction

Symptomatic gallstone disease is one of the most prevalent gastrointestinal conditions worldwide, affecting up to 20% of the population and imposing a substantial clinical and healthcare burden (1, 2).

Laparoscopic cholecystectomy (LC) has become the standard of care, offering clear advantages over open surgery in terms of recovery, length of hospital stay, and patient quality of life (2, 3). Despite its widespread adoption and favorable safety profile, bile duct injury (BDI) remains one of its most severe complications. Although relatively infrequent, its clinical, economic, and medicolegal consequences are considerable (4). Current evidence indicates that misidentification of extrahepatic biliary anatomy is the primary underlying mechanism, often exacerbated by anatomical variability and inflammatory changes, particularly in the acute care setting (5).

To mitigate this risk, several strategies have been developed to improve safety during hepatocystic triangle dissection, including the Critical View of Safety (CVS) (6), standardized anatomical landmarks (7), bailout procedures (8), and intraoperative cholangiography (IOC) (9). More recently, near-infrared fluorescent cholangiography (NIFC) using indocyanine green (ICG) has emerged as an innovative technique that enables real-time visualization of the biliary tree without radiation exposure or direct instrumentation of the bile ducts (10).

This approach has been shown to enhance the identification of key biliary structures and facilitate achievement of the CVS, with potential benefits in reducing complications and improving operative efficiency (11). Its integration into clinical practice is straightforward, with a relatively short learning curve. However, its widespread adoption remains limited by variability in administration protocols, background hepatic fluorescence, and dependence on specialized imaging systems (12).

These limitations are particularly relevant in emergency surgery, where inflammation, anatomical distortion, and time-sensitive decision-making increase procedural complexity and the risk of complications. In this context, fluorescence imaging may provide a meaningful advantage by offering additional real-time anatomical guidance.

This mini-review aims to synthesize current evidence on ICG fluorescent cholangiography in LC, with particular emphasis on its role in acute care surgery. The review also addresses technical principles, clinical impact, current limitations, and key areas of uncertainty that should guide future research.

## 2 Technical foundations of NIFC

### 2.1 Pharmacokinetics, biliary excretion, and safety

ICG is a water-soluble fluorophore belonging to the tricarbocyanine family, with optical properties well suited for near-infrared (NIR) imaging. Following intravenous administration, it rapidly binds to plasma proteins, primarily albumin, and is selectively taken up by hepatocytes. Unlike other contrast agents, ICG undergoes no hepatic metabolism and is excreted exclusively via the biliary system. This unique pharmacokinetic profile, combined with its short plasma half-life, allows predictable pharmacokinetics and enables dynamic intraoperative visualization of the biliary tree (13–15).

Clinically, ICG has a favorable safety profile, with a very low incidence of adverse events. Nevertheless, caution is warranted in selected populations, particularly patients with end-stage renal disease, and known hypersensitivity to ICG or its formulation should be considered a contraindication (16).

### 2.2 Optical principles and image acquisition

NIFC is based on the ability of ICG to absorb NIR light and emit fluorescence detectable by dedicated imaging systems. Because this signal is invisible to the human eye, specialized optical platforms are required (17).

Modern laparoscopic and robotic systems integrate NIR light sources, optical filters, and image-processing software to generate real-time fluorescence images. These can be displayed independently or overlaid onto conventional white-light imaging (WLI), enhancing intraoperative anatomical orientation (18).

NIR light offers greater tissue penetration than visible light, allowing visualization of biliary structures even when partially covered by adipose or inflamed tissue. However, this penetration remains limited (approximately 8–10 mm), particularly in cases of severe inflammation or fibrosis (19).

Overall, these properties enable real-time anatomical mapping, in some cases even prior to dissection, reinforcing the role of fluorescence imaging as a valuable intraoperative decision-making tool (15, 20, 21).

### 2.3 Practical considerations: administration and intraoperative use

ICG can be administered either intravenously or via direct intrabiliary or intracholecystic injection. In clinical practice, the intravenous route remains the preferred approach owing to its simplicity, reproducibility, and seamless integration into standard laparoscopic workflows. Following systemic administration, rapid hepatic uptake and exclusive biliary excretion enable real-time visualization of the extrahepatic biliary tree (15). However, this same pharmacokinetic profile also underlies one of its main limitations: background hepatic fluorescence, which may reduce contrast between biliary structures and surrounding liver parenchyma, particularly when dosing and timing are suboptimal.

Direct intracholecystic injection has been proposed as an alternative strategy to enhance bile duct–to–liver contrast by avoiding hepatic fluorescence (22). Nevertheless, current evidence does not support its routine use. A recent meta-analysis found no significant differences between intravenous and intrabiliary administration in the visualization of key biliary structures. Although intrabiliary injection was associated with reduced hepatic background fluorescence, it resulted in longer operative times and failed to demonstrate superiority in clinically relevant outcomes (23). Moreover, the overall certainty of evidence was very low due to heterogeneity, imprecision, and methodological limitations, precluding firm conclusions.

From a practical standpoint, any theoretical advantage of intrabiliary administration must be weighed against its greater technical complexity. The technique requires gallbladder or duct puncture, precise catheter or needle placement, and careful management of potential dye leakage, all of which may complicate the procedure. In addition, in the presence of cystic duct obstruction, impacted stones, or severe inflammation, contrast distribution may be incomplete or misleading (24). These limitations largely explain why intravenous administration remains the standard approach in most centers.

In practice, NIFC is integrated into multiple stages of the procedure. A structured workflow involves an initial fluorescence assessment prior to dissection to establish a preliminary anatomical roadmap, followed by a second evaluation after exposure of the hepatocystic triangle to confirm the critical anatomy. A final assessment after gallbladder removal allows identification of bile leaks (25). This sequential application is particularly valuable in complex cases, where anatomical relationships evolve throughout the procedure.

Despite ongoing technological advances, fluorescence interpretation remains largely subjective and highly dependent on the surgeon's experience, contributing to interobserver variability (26). In routine practice, fluorescence is assessed qualitatively—based on perceived brightness, contrast, and temporal dynamics—without standardized criteria to define adequate visualization of critical biliary structures. Consequently, similar fluorescence patterns may be interpreted differently, particularly in borderline scenarios.

Quantitative approaches, including bile duct–to–liver ratio (BLR), signal-to-background ratio (SBR), and time–intensity curve analysis, have been proposed to improve objectivity and reproducibility in ICG fluorescence surgery (27, 28). However, their integration into routine clinical practice remains limited due to the lack of standardized acquisition protocols, variability across imaging platforms, and the absence of validated clinically meaningful thresholds.

Furthermore, fluorescence signal characteristics are influenced by multiple factors, including patient-related variables, inflammatory conditions, and technical parameters such as ICG dose and timing, complicating the establishment of universally applicable standards (29). In this context, artificial intelligence (AI) has emerged as a promising tool to enhance image interpretation and standardize intraoperative decision-making, although its clinical applicability still requires robust validation (30).

Overall, the lack of objective, standardized criteria for fluorescence interpretation remains a major barrier to the consistent implementation of NIFC. Consequently, despite technological advances, LC continues to rely largely on the surgeon's subjective interpretation of biliary anatomy.

## 3 Clinical impact

### 3.1 Anatomical identification and intraoperative orientation

Accurate identification of extrahepatic biliary anatomy remains essential for safe LC (31). In this context, NIFC enables dynamic, real-time visualization of biliary structures, facilitating their recognition even in challenging scenarios such as inflammation or anatomical variability (32).

Previous studies have consistently shown that NIFC significantly improves identification rates of key biliary structures compared with conventional WLI (33). More recent high-level evidence further reinforces this advantage. A contemporary integrative analysis incorporating randomized controlled trials and meta-analyses demonstrated that NIFC significantly increases identification rates of the common bile duct and common hepatic duct compared with WLI, with pooled relative risks of 1.68 and 1.81, respectively (34). These findings suggest that fluorescence provides a more reliable anatomical roadmap, particularly during the early phases of dissection.

However, these benefits should be interpreted with caution. Improved identification of biliary structures represents a surrogate endpoint, and its direct translation into improved clinical outcomes remains uncertain. In addition, substantial heterogeneity across studies limits the generalizability of these results. In highly complex cases—such as severe inflammation or Mirizzi syndrome—performance may be limited by reduced tissue penetration or impaired biliary flow (35, 36).

A key advantage of NIFC is its ability to provide anatomical information prior to dissection, allowing the surgeon to establish a preliminary roadmap and potentially reduce the risk of misidentification. This feature distinguishes NIFC from IOC, which typically requires prior dissection and cannulation of the cystic duct.

From a comparative perspective, IOC has traditionally been considered the reference standard for intraoperative biliary imaging, given its ability to provide high-resolution radiographic visualization, including intrahepatic ducts. However, its routine use is limited by technical complexity, the need for cystic duct cannulation, radiation exposure, and reliance on additional equipment and personnel. Furthermore, IOC is not universally feasible, particularly in cases of cystic duct obstruction or severe inflammation, where cannulation may be difficult or unsafe.

In contrast, NIFC offers a non-invasive, repeatable, and workflow-compatible imaging modality that can be applied throughout the procedure without interrupting surgical flow. Available data suggest that NIFC achieves similar visualization rates of the cystic duct, common bile duct, and their junction, with a potential advantage in identifying the common hepatic duct (11). Nevertheless, these findings should be interpreted cautiously, given the heterogeneity of the available evidence.

Importantly, both modalities have inherent limitations that must be critically acknowledged. The performance of NIFC is constrained by the limited penetration of NIR light, which may reduce signal quality in patients with obesity, fibrosis, or severe inflammation. Additionally, background hepatic fluorescence can impair contrast when ICG administration is not optimized. Conversely, while IOC is less affected by tissue characteristics and allows visualization of intrahepatic ducts, it remains technically demanding and is associated with a non-negligible failure rate.

A critical and often underrecognized aspect is that both NIFC and IOC remain dependent on surgeon interpretation. While IOC is traditionally perceived as more objective, misinterpretation of cholangiographic images represents a significant contributor to BDI. Similarly, NIFC relies on qualitative assessment of fluorescence patterns, which introduces interobserver variability, particularly in borderline or complex scenarios. Thus, neither technique fully eliminates the risk of anatomical misidentification.

Overall, current evidence supports NIFC as a valuable adjunct for improving intraoperative orientation and facilitating anatomical recognition, particularly during the learning curve, where it enhances anatomical interpretation and surgical confidence (37). However, it should not be considered a substitute for established safety principles.

Ultimately, despite advances in imaging technologies, LC continues to rely fundamentally on the surgeon's ability to interpret biliary anatomy accurately. In this context, fluorescence imaging enhances—but does not replace—sound surgical judgment and adherence to the CVS.

### 3.2 Impact on surgical complications

The potential of NIFC to reduce surgical complications has received increasing attention, particularly because misidentification of biliary anatomy remains a primary mechanism underlying BDI. From a mechanistic perspective, improved visualization of the extrahepatic biliary tree should facilitate safer dissection and reduce the risk of anatomical misinterpretation. However, the extent to which this anatomical advantage translates into a measurable reduction in clinically relevant complications remains uncertain.

Several observational studies and meta-analyses have suggested lower rates of BDI, conversion to open surgery, and perioperative complications when NIFC is used. Nevertheless, these findings should be interpreted with caution, as the low incidence of BDI limits statistical power and precludes definitive conclusions (38, 39). Consistently, recent comparative evidence has demonstrated improved visualization of the common bile duct and common hepatic duct with NIFC vs. WLI, but without a statistically significant reduction in BDI or conversion rates (34). Notably, trial sequential analyses indicate that extremely large sample sizes would be required to adequately assess the impact of NIFC on such rare but clinically critical outcomes.

Beyond BDI, some studies have reported an association between fluorescence quality and both intraoperative and postoperative complications, suggesting that suboptimal fluorescence may reflect increased surgical complexity (29). In this context, NIFC may act as an indirect indicator of surgical difficulty.

Current evidence therefore supports NIFC as a valuable tool that may enhance intraoperative safety through improving anatomical orientation, but not as an independent guarantee against complications. When fluorescence is weak, incomplete, or discordant with the operative findings, surgeons should avoid proceeding with potentially unsafe dissection and instead rely on established safety strategies.

### 3.3 Influence on operative time and hospital stay

The effect of fluorescence imaging on operative time and length of stay remains uncertain. While initially thought to prolong procedures, current evidence suggests that it may reduce operative time by facilitating faster and more accurate anatomical identification (29, 40).

This benefit may be particularly relevant in complex cases, where fluorescence can support the achievement of the CVS and reduce the need for bailout procedures or conversion to open surgery (38, 41).

Data regarding hospital stay are limited, although some studies suggest a potential reduction, likely related to fewer complications and improved postoperative recovery (29). Overall, while fluorescence imaging may improve procedural efficiency, these benefits have not been consistently demonstrated.

## 4 Role in acute care surgery

Given its role in improving intraoperative anatomical orientation, the potential value of NIFC becomes particularly relevant in emergency settings, where LC is inherently more complex.

In acute cholecystitis, inflammation, edema, adhesions, and distortion of the hepatocystic triangle significantly impair anatomical recognition and increase the risk of biliary misidentification and BDI (8, 42).

In this context, the utility of NIFC is amplified in situations of increased anatomical uncertainty, where even partial visualization of biliary structures may support safer decision-making. Its main advantage lies in enhancing real-time visualization of the biliary tree often prior to dissection which is particularly valuable in inflamed or anatomically distorted fields (43). Available clinical evidence is supportive but not definitive. A prospective case-control study in emergency LC demonstrated a significantly lower conversion rate to open surgery and shorter hospital stay with the use of ICG, although differences in BDI and overall complications did not reach statistical significance (44). Similarly, recent meta-analyses in difficult LC have reported reductions in operative time, conversion rates, and length of stay, without consistent differences in postoperative complications (45).

However, these findings must be interpreted cautiously. The available evidence is highly heterogeneous, with variability in disease severity, definitions of difficult cholecystectomy, ICG administration protocols, and surgeon experience. Moreover, improvements in surrogate outcomes—such as operative time or conversion rates—do not necessarily translate into a proportional reduction in major complications such as BDI.

Importantly, the performance of NIFC may be compromised in the very scenarios where it is most needed. Severe inflammation, fibrosis, obesity, or cystic duct obstruction can reduce fluorescence penetration or impair biliary excretion, resulting in weak or potentially misleading signals (46).

From a guideline perspective, international consensus statements support the use of fluorescence imaging as a safe adjunct in emergency biliary surgery, particularly to facilitate anatomical orientation and achievement of the CVS (15, 21). However, current evidence does not support its routine use as a standard of care in all emergency cases.

Taken together, NIFC plays a complementary and context-dependent role in acute care LC. It enhances intraoperative orientation and may reduce the need for bailout strategies in complex cases, but it does not eliminate the inherent challenges of inflamed biliary anatomy. As such, fluorescence imaging should be considered an adjunct to—rather than a replacement for—sound surgical judgment and strict adherence to established safety principles.

## 5 Limitations, controversies and determinants of effectiveness

Despite its growing adoption and intraoperative utility, NIFC remains subject to relevant limitations that affect its effectiveness, reproducibility, and generalizability. Importantly, many of these limitations are closely linked to both modifiable and non-modifiable determinants of fluorescence performance, underscoring the need for a more integrated and standardized approach (28, 46).

A key unresolved issue is the lack of consensus regarding the optimal dose and timing of ICG administration. Substantial heterogeneity exists across studies, ranging from high-dose preoperative protocols to low-dose or intraoperative strategies (12, 47). Emerging evidence suggests that ultra-low intraoperative dosing may achieve comparable biliary visualization while reducing hepatic background fluorescence and simplifying workflow (48, 49). However, variability in imaging systems, patient characteristics, and study design currently precludes the establishment of standardized protocols.

Background hepatic fluorescence remains a major limitation, as it can obscure critical biliary structures and reduce contrast, particularly when pharmacokinetic parameters are suboptimal. This reflects the intrinsic lack of biliary specificity of ICG, whose signal initially distributes within the vascular and hepatic compartments before biliary excretion, potentially compromising interpretation in inflamed or steatotic livers (28).

In addition, fluorescence quality is significantly influenced by patient- and disease-related factors. Obesity, inflammation, fibrosis, and underlying liver dysfunction may attenuate signal intensity or delay biliary excretion, reducing visualization quality (29). Notably, these are the scenarios in which enhanced anatomical guidance is most needed, highlighting a fundamental limitation of current fluorescence technology (21, 50).

Technical variability further impacts performance. Differences in imaging platforms, camera sensitivity, and processing algorithms can affect fluorescence detection and image quality (49). Although technological advances have improved visualization even with lower ICG doses, they also introduce variability across centers and limit reproducibility.

A critical limitation is the fundamentally qualitative nature of fluorescence interpretation. NIFC remains highly dependent on subjective intraoperative assessment, with no universally accepted criteria to define adequate visualization. This contributes to interobserver variability and limits consistency. Although quantitative metrics such as BLR or SBR have been proposed, their clinical implementation remains limited due to the lack of standardized protocols and validated thresholds. Emerging AI-based tools may improve interpretation, although validation remains limited (30).

From a physical standpoint, the limited tissue penetration of near-infrared light constrains performance in cases of severe inflammation or fibrosis and precludes reliable visualization of intrahepatic ducts (28). Furthermore, unlike IOC, NIFC does not provide intraluminal ductal imaging and therefore cannot reliably detect choledocholithiasis (30).

These limitations have stimulated the development of next-generation fluorophores with improved biliary specificity. Preclinical studies suggest that novel agents may provide faster pharmacokinetics, higher target-to-background ratios, and improved performance under inflammatory conditions (51). However, these technologies remain preclinical or early-stage and require clinical validation before adoption.

In summary, while NIFC represents a meaningful advancement in image-guided biliary surgery, its current limitations remain closely intertwined with the determinants of its effectiveness. Its broader consolidation in routine practice will depend on the standardization of administration protocols, optimization of imaging technologies, and the development of objective frameworks for fluorescence interpretation. Until then, fluorescence imaging should be regarded as a powerful adjunct that enhances—but does not replace—the surgeon's critical judgment in the interpretation of biliary anatomy.
